# Impact of ambient temperature on respiratory disease: a case-crossover study in Seoul

**DOI:** 10.1186/s12931-024-02699-0

**Published:** 2024-02-05

**Authors:** Hyewon Lee, Hee-Young Yoon

**Affiliations:** 1 Department of Health Administration and Management, College of Medical Sciences, Soonchunhyang University, Asan, Republic of Korea; 2 Department of Software Convergence, Soonchunhyang University Graduate School, Asan, Republic of Korea; 3 Division of Allergy and Respiratory Diseases, Department of Internal Medicine, Soonchunhyang University Seoul Hospital, 59 Daesagwanro, Yongsan-gu, Seoul, 04401 Republic of Korea

## Abstract

**Background:**

Respiratory diseases contribute to global morbidity and mortality, and temperature is a significant factor. We investigated the association between ambient temperature and emergency department (ED) visits for various respiratory diseases in Seoul, South Korea.

**Methods:**

Using data from the National Emergency Department Information System (2008–2017), we analysed 1,616,644 ED visits for respiratory diseases, categorised according to the Korean Standard Classification of Diseases 7th revision codes (J00-J99). Using a time-stratified case-crossover design and a distributed lag nonlinear model, we investigated the effect of temperature exposure on ED visits for respiratory diseases, calculating the relative risk (RR) for the maximum risk temperature (MaxRT) of both cold and hot extremes compared to the minimum risk temperature (MinRT).

**Results:**

Cold temperatures (MaxRT: -9.0 °C) resulted in a 2.68-fold increase (RR = 2.68, 95% CI = 2.26–3.14) in ED visits for total respiratory diseases, while hot temperatures (MaxRT: 29.4 °C) led to a 1.26-fold increase (RR = 1.26, 95% CI = 1.11–1.42) compared to the MinRT (24.8 °C). Cold temperatures increased the risk of most respiratory diseases, except interstitial lung disease, whereas hot temperatures increased ED visits for acute upper respiratory infections and influenza. Cold temperatures increased ED visits for all age groups, especially those aged 18–64 (RR = 3.54, 95% CI = 2.90–4.33), while hot temperatures significantly affected those < 18 (RR = 1.45, 95% CI = 1.27–1.66). The risk levels were similar in both males and females, regardless of hot and cold temperatures.

**Conclusion:**

Our findings underscore the significant impact of both cold and heat exposure on ED visits for respiratory diseases, with varying intensities and risk profiles across different population groups.

**Supplementary Information:**

The online version contains supplementary material available at 10.1186/s12931-024-02699-0.

## Background

Respiratory diseases are a significant global health challenge that contribute to high morbidity and mortality rates worldwide. In 2019, chronic respiratory diseases, such as chronic obstructive pulmonary disease (COPD), asthma, and other respiratory conditions, were the third leading cause of death globally, resulting in 4.0 million deaths and affecting 454.6 million individuals [1, 2]. These diseases also impose a substantial burden on healthcare systems owing to hospitalisations, emergency department visits, and long-term care expenses [3–6]. Furthermore, respiratory infections, including upper and lower respiratory tract infections, contribute to a substantial global burden, with billions of cases and millions of deaths reported annually [7, 8].

Weather conditions, particularly temperature, significantly contribute to the development of respiratory diseases. Understanding the relationship between temperature and respiratory diseases is crucial, particularly given the increasing frequency and intensity of extreme temperature events caused by climate change [9]. Previous studies have demonstrated that both heat and cold exposure are associated with an increased risk of respiratory symptoms, exacerbation, and hospitalization [10–22]. However, most of these studies have focused on specific respiratory diseases in relation to either heat or cold exposure, highlighting the need for more comprehensive research examining the impact of both heat and cold on overall respiratory health. Thus, this study aimed to investigate the association between heat and cold exposure and emergency department (ED) visits for respiratory diseases in Seoul, South Korea, using a comprehensive national database. We hypothesised that both cold and heat exposure would affect respiratory diseases, with the intensity of the effects and risk groups varying across different diseases.

## Methods

### Study design and data extraction

We used data from the National Emergency Department Information System (NEDIS), a comprehensive database established by the National Emergency Medical Center in 2003. The NEDIS contains detailed information on patient demographics, emergency department visits, diagnoses, treatments, and outcomes from approximately 98% of all emergency medical facilities in South Korea. Our study was conducted in accordance with the Declaration of Helsinki and the study protocol was approved by the Institutional Review Board of Soonchunhyang University (202,108-SB-076-01). As the NEDIS database contains de-identified information, informed consent was not required.

### Study populations and primary outcome

Our study population consisted of patients who visited the ED in Seoul between 2008 and 2017 with a primary diagnosis of respiratory disease. These diseases were classified based on the Korean Standard Classification of Diseases 7th revision codes J00–J99, a modification of the International Classification of Diseases 10th revision, to suit the specific health system in South Korea. The diseases were further categorised into specific groups: acute upper respiratory infections (AURI) (J00-J06), influenza and pneumonia (J09-J18), acute lower respiratory infection (ALRI) (J20-J22), chronic lower respiratory diseases (CLRD) (J40-J47), COPD (J42-J44 except for J43 alone), asthma (J45), acute respiratory distress syndrome (ARDS) and pulmonary edema (J80, J81), interstitial lung disease (ILD) (J84), and pneumothorax (J93). The primary outcome of interest was all ED visits with respiratory disease codes assigned as the primary diagnosis at discharge.

### Exposure assessment

This study focused on assessing the exposure to daily ambient temperature in Seoul, the capital and largest city in South Korea. To estimate this parameter, we collected hourly ambient temperature data from Seoul’s weather monitoring station using the Automated Surface Observing System of the Korea Meteorological Administration. We calculated the 24-hour mean, maximum, and minimum temperatures for each day using hourly data.

Seoul is situated within a temperate zone at 37.57°N and 126.98°E. The city experiences four distinct seasons, characterised by hot summers with an average maximum temperature of 32 °C, allowing for the examination of heat exposure’s effects on respiratory health. Additionally, Seoul’s winters are cold, with an average minimum temperature of -15 °C, making it an ideal setting to study the impact of cold exposure on respiratory diseases. Seoul also experiences a rainy season in July and August, which potentially influences variations in air quality and respiratory conditions during this period. The city’s substantial population and diverse temperature range make it a suitable location for assessing the association between ambient temperature and respiratory diseases.

### Statistical analysis

This time-stratified case-crossover study used conditional Poisson regression to investigate the impact of daily mean temperature on ED visits for respiratory diseases. For each patient, corresponding 3 ~ 4 control days were meticulously selected from the same year, month, and day of the week stratum, ensuring a comparable day without an ED visit. This design allowed us to effectively control for slowly varying factors such as sex, genetic predisposition, lifestyle, socioeconomic status, and comorbidities. By comparing the temperatures on case and control days, we aimed to isolate the specific impact of temperature on ED visits.

To assess both cold and hot temperature impacts, we conducted a conditional Poisson regression with a distributed lag nonlinear model (DLNM), which allowed us to construct a flexible temperature structure, capturing the potential nonlinear and lagged effects of exposure. The model was constructed using quadratic B-splines with two internal knots (33.3rd and 63.7th) for exposure-response and natural cubic B-splines with three knots at equal intervals in log-scale for lag-response, guided by the Quasi-Akaike information criteria and previous studies (Additional file 1: Table S1) [23, 24]. Using the DLNM, we obtained the minimum risk temperature (MinRT) and maximum risk temperature (MaxRT), along with the corresponding percentiles (%), providing insights into the temperature range associated with the highest and lowest risks. We calculated the relative risks (RRs) for MaxRT compared to MinRT using a lag structure of up to 21 days for the main temperature effect after adjusting for holidays, influenza epidemic, and 2-day moving averages (lag 0–1) of relative humidity and barometric pressure.

We conducted subgroup analyses based on age (< 18 years, 18–64 years, and ≥ 65 years) and sex (male vs. female). Owing to the rarity of COPD and ILD in individuals under 18 years of age, a subanalysis for this age group was not conducted. To enhance our findings’ robustness, we performed four sensitivity analyses: (1) using the minimum and maximum temperature, (2) exploring different lag periods (0–7, 0–14, and 0–28 days), (3) adjusting for ambient pollutants, such as particulate matter 10 and 2.5 micrometers or less in diameter, nitrogen dioxide, ozone, sulfur dioxide, and carbon monoxide, and (4) exploring various model specifications for the exposure-response relationship, including different combinations of internal knots and changing the spline function from quadratic to cubic [25]. All statistical analyses were performed using SAS version 9.4 (SAS Institute Inc., Cary, NC) and R version 3.5.3 (R Core Team, 2019).

## Results

### Baseline characteristics

Between 2008 and 2017, 1,616,644 ED visits for respiratory diseases were recorded in Seoul, South Korea (Table 1). The average number of daily ED visits for respiratory diseases was 442.6, with AURI (253.5) being the most common diagnosis, followed by influenza and pneumonia (100.4), ALRI (28.4), and CLRD (27.4). Seoul’s average temperature during this period was 12.9 °C, with temperatures extremes ranging from − 18.0 to 36.4 °C.

**Table 1:** Descriptive statistics for daily ED visits for respiratory diseases and levels of environmental variables in Seoul, South Korea, 2008–2017

Variables	Mean	SD	Min	Q2	Median	Q3	Max
ED visits							
Total respiratory diseases	442.6	329.5	105	276	352	498	4336
Acute upper respiratory infection	253.5	179.3	52	150	199	286	2018
Influenza and pneumonia	100.4	166.4	6	38	61	94	2331
Acute lower respiratory infection	28.4	19.6	1	15	24	36	178
Chronic lower respiratory diseases	27.4	12.8	3	19	25	34	108
COPD	5.7	3.2	0	3	5	8	22
Asthma	11.0	5.6	0	7	10	14	52
ARDS and pulmonary oedema	1.2	1.5	0	0	1	2	12
ILD	1.6	1.5	0	1	1	2	11
Pneumothorax	8.8	3.7	0	6	9	11	25
Environmental variables							
Mean temperature (℃)	12.9	10.8	-14.6	3.5	14.5	22.7	31.8
Maximum temperature (℃)	17.2	11.0	-11.2	7.7	19.4	27.0	36.4
Minimum temperature (℃)	9.1	10.9	-18.0	-0.3	10.3	18.8	28.7
Humidity (%)	59.9	14.9	18.3	49.0	60.0	70.5	99.4
Air pressure (hPa)	1016.2	8.2	989.9	1009.7	1016.4	1022.7	1038.1
PM_10_ (µg/m^3^)	48.5	28.9	4.6	30.8	43.0	58.9	568.7
PM_2.5_ (µg/m^3^)	24.6	13.4	3.3	15.3	22.0	30.4	121.6
NO_2_ (ppb)	35.1	12.2	9.0	25.8	33.5	43.1	82.0
SO_2_ (ppb)	5.3	1.8	2.4	4.0	4.8	6.0	21.7
CO (0.1ppm)	6.5	2.6	2.3	4.7	5.8	7.5	25.5
O_3_ (ppb)	31.4	16.8	2.1	18.9	28.9	42.2	105.4

ED, emergency department; COPD, chronic obstructive pulmonary disease; ARDS, acute respiratory distress syndrome; ILD, interstitial lung disease; PM_10_, particles *<* 10 μm in diameter; PM_2.5_, particles *<* 2.5 μm in diameter; NO_2_, nitrogen dioxide; SO_2_, sulfur dioxide; CO, carbon monoxide; O_3_, ozone; SD, standard deviation; Min, minimum; Q2, quartile 2, Q3, quartile 3; Max, maximum

### The association between mean temperature and risk of ED

In our analysis, the MinRT for total respiratory diseases was 24.8 °C, corresponding to the 85.5th percentile (%). Similar MinRT values were observed for AURI (24.3 °C, 82.5%), influenza (25.0 °C, 86.5%), and ILD (26.4 °C, 92.4%) (Table 2). All other disease categories also reported MinRT values at or above the 80%, except for pneumothorax, which had a MinRT of 14.0 °C (48.8%).

**Table 2:** Associations between mean ambient temperature and ED visits for respiratory diseases

	MinRT (%)	Cold temperature		Hot temperature	
		MaxRT (%)	RR (95% CI)	MaxRT (%)	RR (95% CI)
Total respiratory diseases	24.8 (85.5)	-9.0 (1.0)	2.68 (2.26, 3.17)*	29.4 (99.0)	1.26 (1.11, 1.42)*
Acute upper respiratory infection	24.3 (82.5)	-9.0 (1.0)	2.00 (1.68, 2.39)*	29.4 (99.0)	1.23 (1.08, 1.40)*
Influenza and pneumonia	25.0 (86.5)	-9.0 (1.0)	11.23 (8.83, 14.28*)	29.4 (99.0)	1.64 (1.31, 2.06)*
Acute lower respiratory infection	28.0 (96.8)	10.7 (41.3)	2.01 (1.72, 2.35)*	29.4 (99.0)	1.01 (0.95, 1.07)
Chronic lower respiratory diseases	29.4 (99.0)	10.0 (39.7)	1.53 (1.29, 1.80)*	NA	
COPD	23.9 (80.7)	-9.0 (1.0)	1.54 (1.11, 2.13)*	29.4 (99.0)	1.06 (0.84, 1.34)
Asthma	29.4 (99.0)	12.5 (45.3)	1.88 (1.49, 2.37)*	NA	
ARDS and pulmonary oedema	23.8 (80.1)	-7.9 (1.0)	1.86 (1.00, 3.45)*	29.4 (99.0)	1.31 (0.81, 2.12)
ILD	26.4 (92.4)	7.4 (34.8)	1.30 (0.86, 1.97)	29.4 (99.0)	1.02 (0.77, 1.36)
Pneumothorax	14.0 (48.8)	-5.2 (4.9)	1.18 (1.00, 1.39)*	29.4 (99.0)	1.22 (0.98, 1.52)

ED, emergency department; RR, relative risk; CI, confidence interval; MinRT, minimum risk temperature; MaxRT, maximum risk temperature; COPD, chronic obstructive pulmonary disease; ARDS, acute respiratory distress syndrome; ILD, interstitial lung disease; NA, not available

*p < 0.05

**Fig. 1:**
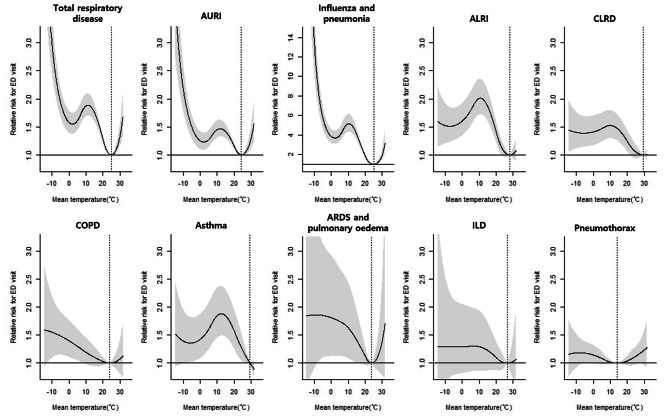
Overall cumulative associations between mean ambient temperature and emergency department visits for subtypes of respiratory diseases over 0–21 lag days. Dotted vertical lines indicate MinRT, while the solid line with shaded areas represents the relative risk with 95% confidence intervals. ED, emergency department; MinRT, minimum risk temperature; AURI, acute upper respiratory infection; ALRI, acute lower respiratory infection; CLRD, chronic lower respiratory disease; COPD, chronic obstructive pulmonary disease; ARDS, acute respiratory distress syndrome; ILD, interstitial lung disease

**Fig. 2:**
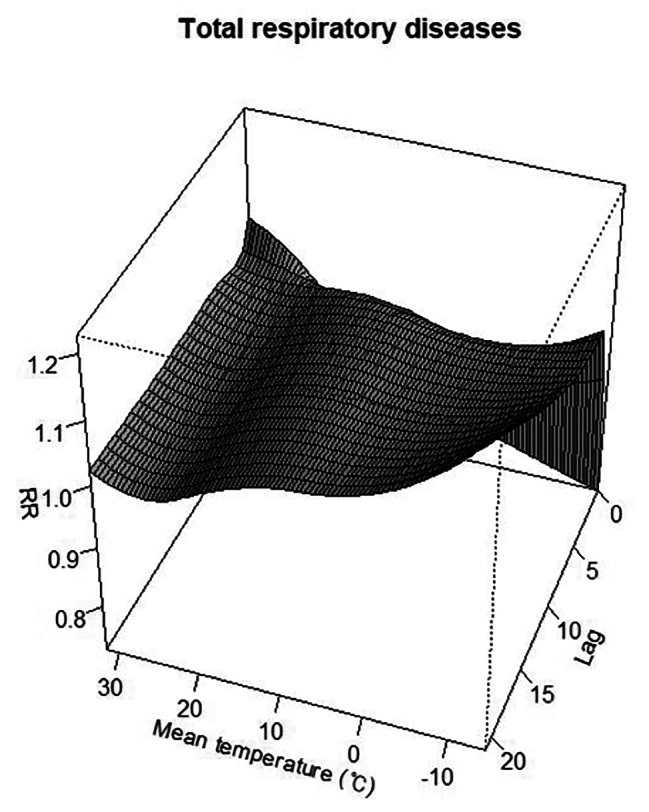
Three-dimensional curves for the RRs of emergency department visits for total respiratory diseases with mean ambient temperature over 0–21 lag days. The x-axis represents the mean ambient temperature, y-axis represents the lag days, and z-axis represents the relative risk of emergency department visits. Each curve represents a different dataset. The height and colour of the curves indicate the level of risk, with higher and darker curves representing a higher risk. RRs, relative risks

In the cold temperatures, we observed a significant 2.68-fold increase in ED visits (95% CI = 2.26–3.14) for all respiratory diseases at a MaxRT of -9.0 °C (Figs. 1 and 2). This MaxRT was consistently associated with increased ED visit risk for AURI (RR = 1.68, 95% CI = 1.68–2.39), influenza and pneumonia (RR = 11.23, 95% CI = 8.83–14.28), and COPD (RR = 1.54, 95% CI = 1.11–2.13). ARDS and pulmonary oedema (RR = 1.86, 95% CI = 1.00-3.45) and pneumothorax (RR = 1.18, 95% CI = 1.00-1.39) showed similar trends with MaxRTs of -7.9 °C and − 5.2 °C, respectively. Meanwhile, ALRI (RR = 2.01, 95% CI = 1.72–2.35), CLRD (RR = 1.53, 95% CI = 1.29–1.80), and asthma (RR = 1.88, 95% CI = 1.49–2.37) were significantly linked to ED visits at colder temperatures, specifically when MaxRT was above 10.0 °C.

Hot temperatures (MaxRT: 29.4 °C) were significantly associated with a 1.26-fold increase (95% CI = 1.11–1.42) in ED visits for total respiratory diseases. Similar trends were observed for AURI (RR = 1.23, 95% CI = 1.08–1.40) and influenza and pneumonia (RR = 11.23, 95% CI = 8.83–14.28). However, no significant associations were observed between hot temperatures and other respiratory diseases.

### Subgroup analysis stratified by age

**Fig. 3:**
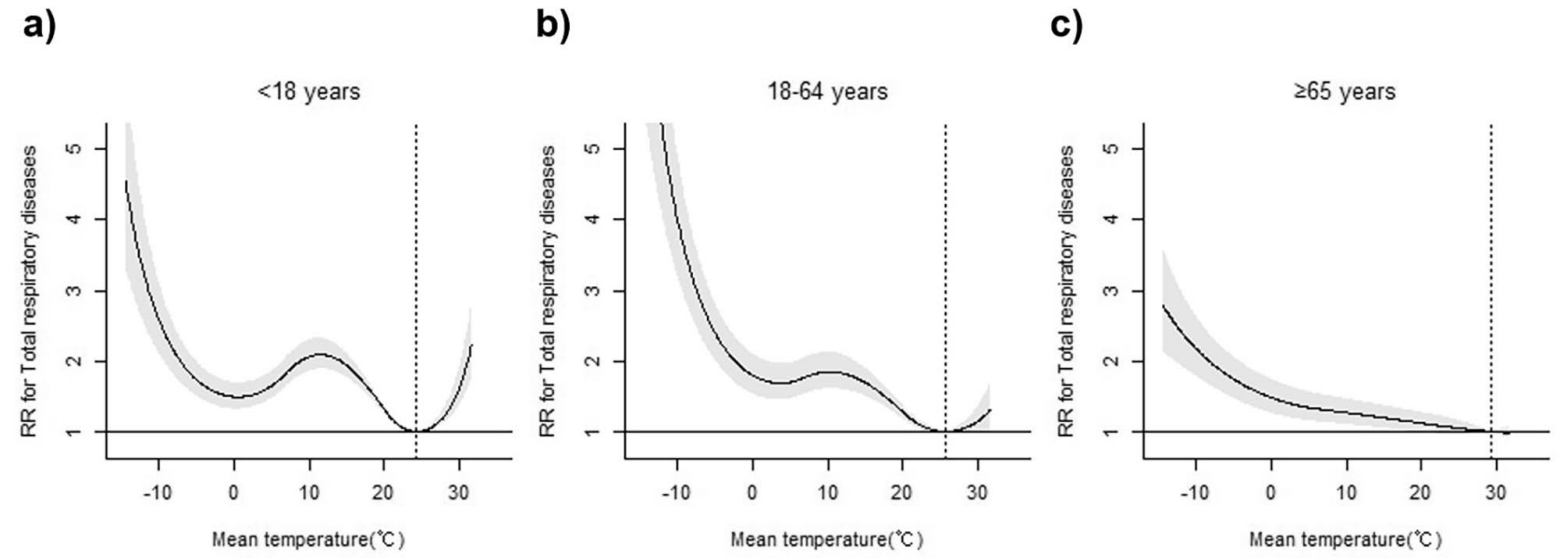
Overall cumulative associations between mean ambient temperature and emergency department visits for total respiratory diseases, stratified by age. (**a**) individuals under 18 years of age, (**b**) individuals between 18 and 64 years of age, (**c**) individuals aged 65 years and above. Dotted vertical lines indicate MinRT, while the solid line with shaded areas represents the relative risk with 95% confidence intervals. ED, emergency department; MinRT, minimum risk temperature

In the age-stratified subgroup analysis, cold temperatures (MaxRT: -9.0 °C) were associated with increased ED visits for total respiratory diseases across all age groups, with the highest RR observed in the 18–64 age group (RR = 3.54, 95% CI = 2.90–4.33) compared to those aged < 18 years (RR = 2.34, 95% CI = 1.95–2.80) and ≥ 65 years (RR = 2.07, 95% CI = 1.71–2.49) (Table 3; Fig. 3). Cold temperatures were significantly associated with increased ED visits for AURI, influenza, and ALRI in all age groups. Cold temperatures were significantly associated with CLRD, asthma, and ARDS and pulmonary oedema in the group aged 18–64 and ≥ 65 years. For individuals aged ≥ 65 years, significant associations were observed between cold temperature and COPD and pneumothorax.

**Table 3:** Age-stratified associations between mean ambient temperature and ED visits for respiratory diseases

Age groups	MinRT (%)	Cold temperature		Hot temperature	
		MaxRT (%)	RR (95% CI)	MaxRT (%)	RR (95% CI)
Total respiratory diseases					
<18 years	24.4 (83.1)	-9.0 (1.0)	2.34 (1.95, 2.80)*	29.4 (99.0)	1.45 (1.27, 1.66)*
18–64 years	25.8 (90.0)	-9.0 (1.0)	3.54 (2.90, 4.33)*	29.4 (99.0)	1.10 (0.97, 1.26)
≥65 years	29.4 (99.0)	-9.0 (1.0)	2.07 (1.71, 2.49)*	NA	
Acute upper respiratory infection					
<18 years	23.8 (80.1)	-9.0 (1.0)	2.83 (2.08, 3.85)*	29.4 (99.0)	1.39 (1.22, 1.57)*
18–64 years	27.1 (94.8)	-9.0 (1.0)	5.01 (3.57, 7.03)*	29.4 (99.0)	1.02 (0.93, 1.13)
≥65 years	29.4 (99.0)	-9.0 (1.0)	6.19 (3.42, 11.21)*	NA	
Influenza and pneumonia					
<18 years	24.7 (85.1)	-9.0 (1.0)	16.10 (12.32, 21.02)*	29.4 (99.0)	2.33 (1.73, 3.15)*
18–64 years	24.6 (84.5)	-9.0 (1.0)	20.07 (14.69, 27.43)*	29.4 (99.0)	2.00 (1.43, 2.81)*
≥65 years	29.4 (99.0)	-9.0 (1.0)	2.14 (1.66, 2.76)*	NA	
Acute lower respiratory infection					
<18 years	27.9 (96.7)	11.2 (42.5)	2.10 (1.78, 2.47)*	29.4 (99.0)	1.01 (0.95, 1.09)
18–64 years	29.4 (99.0)	-9.0 (1.0)	4.42 (2.62, 7.45)*	NA	
≥65 years	14.9 (51.5)	-9.0 (1.0)	1.68 (1.00, 2.83)*	29.4 (99.0)	1.20 (0.63, 2.29)
Chronic lower respiratory diseases					
<18 years	-9.0 (1.0)	NA		12.5 (45.3)	2.00 (1.55, 2.60)*
18–64 years	29.4 (99.0)	-9.0 (1.0)	2.07 (1.49, 2.89)*	NA	
≥65 years	24.1 (81.5)	-5.8 (4.2)	1.65 (1.33, 2.06)*	29.4 (99.0)	1.07 (0.87, 1.30)
COPD					
<18 years^†^					
18–64 years	29.4 (99.0)	-9.0 (1.0)	2.01 (0.87, 4.66)	NA	
≥65 years	24.2 (82.0)	-8.5 (1.4)	1.50 (1.06, 2.14)*	29.4 (99.0)	1.56 (0.88, 2.77)
Asthma					
<18 years	-8.0 (1.9)	-9.0 (1.0)	1.00 (0.94, 1.07)	14.3 (49.4)	3.01 (2.09, 4.34)*
18–64 years	29.4 (99.0)	11.4 (43.0)	2.03 (1.47, 2.82)*	NA	
≥65 years	26.8 (93.7)	-9.0 (1.0)	1.91 (1.14, 3.21)*	29.4 (99.0)	1.01 (0.79, 1.29)
ARDS and pulmonary oedema					
<18 years	0.1 (15.9)	-9.0 (1.0)	2.66 (0.44, 16.26)	27.9 (96.7)	5.39 (1.00, 29.00)*
18–64 years	22.8 (75.3)	2.9 (23.5)	2.23 (1.07, 4.63)*	29.4 (99.0)	2.12 (0.91, 4.98)
≥65 years	25.1 (87.1)	-9.0 (1.0)	2.61 (1.19, 5.73)*	29.4 (99.0)	1.11 (0.69, 1.79)
ILD					
<18 years^†^					
18–64 years	-9.0 (1.0)	NA		29.4 (99.0)	1.34 (0.40, 4.44)
≥65 years	29.4 (99.0)	-9.0 (1.0)	1.60 (0.67, 3.80)	NA	
Pneumothorax					
<18 years	-9.0 (1.0)	NA		27.1 (94.8)	1.61 (0.84, 3.05)
18–64 years	14.2 (49.2)	-4.8 (5.5)	1.16 (0.95, 1.42)	29.4 (99.0)	1.10 (0.83, 1.44)
≥65 years	22.0 (72.1)	-9.0 (1.0)	2.41 (1.22, 4.79)*	29.4 (99.0)	1.82 (1.08, 3.08)*

ED, emergency department; RR, relative risk; CI, confidence interval; MinRT, minimum risk temperature; MaxRT, maximum risk temperature; COPD, chronic obstructive pulmonary disease; ARDS, acute respiratory distress syndrome; ILD, interstitial lung disease; NA, not available

*p < 0.05

^†^No analysis for individuals under 18 years old due to low occurrence

Hot temperatures significantly increased ED visits for total respiratory diseases in individuals < 18 years (RR = 1.45, 95% CI = 1.27–1.66) (Table 3). This association extended to all sub-respiratory diseases, except pneumothorax, which showed a significant association in patients aged ≥ 65 years. The MaxRT values for these associations, ranging from 27.9 to 29.4 °C, fell within the 96.7 to 99.0% range.

### Subgroup analysis stratified by sex

**Fig. 4:**
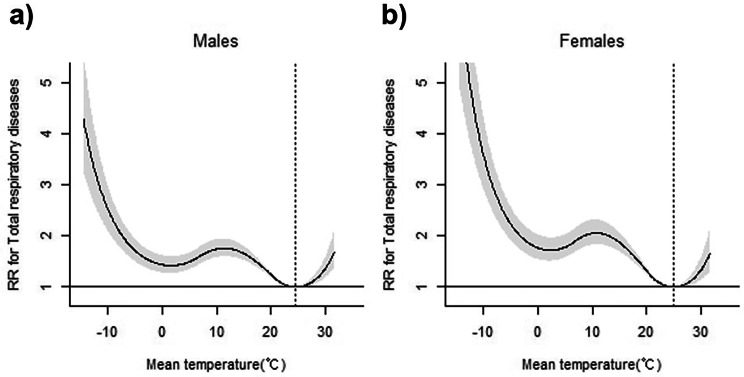
Overall cumulative associations between mean ambient temperature and emergency department visits for total respiratory diseases, stratified by age and sex. (**a**) males, and (**b**) females. Dotted vertical lines indicate MinRT, while the solid line with shaded areas represents the relative risk with 95% confidence intervals. ED, emergency department; MinRT, minimum risk temperature

In the subgroup analysis stratified by sex, both males and females demonstrated an increased RR for total respiratory disease ED visits at colder temperatures (MaxRT: − 9 °C, 1.0%), with RRs of 2.29 (95% CI = 1.95–2.70) and 3.19 (95% CI = 2.66–3.83), respectively (Table 4; Fig. 4). Similar patterns were observed for AURI, influenza, ALRI, and CLRD. However, for COPD, ARDS and pulmonary oedema, and pneumothorax, males had a higher risk at lower temperatures, whereas females had a higher risk for asthma at colder temperatures.

**Table 4:** Sex-stratified associations between mean ambient temperature and ED visits for respiratory diseases

Sex groups	MinRT (%)	Cold temperature		Hot temperature	
		MaxRT (%)	RR (95% CI)	MaxRT (%)	RR (95% CI)
Total respiratory diseases					
Males	24.6 (84.5)	-9.0 (1.0)	2.29 (1.95, 2.70)*	29.4 (99.0)	1.27 (1.12, 1.43)*
Females	25.0 (86.5)	-9.0 (1.0)	3.19 (2.66, 3.83)*	29.4 (99.0)	1.24 (1.08, 1.42)*
Acute upper respiratory infection					
Males	24.1 (81.5)	-9.0 (1.0)	1.80 (1.51, 2.16)*	29.4 (99.0)	1.26 (1.11, 1.44)*
Females	24.7 (85.1)	-9.0 (1.0)	2.24 (1.86, 2.71)*	29.4 (99.0)	1.20 (1.04, 1.37)*
Influenza and pneumonia					
Males	24.7 (85.1)	-9.0 (1.0)	16.10 (12.32, 21.02)*	29.4 (99.0)	2.33 (1.73, 3.15)*
Females	24.6 (84.5)	-9.0 (1.0)	20.07 (14.69, 27.43)*	29.4 (99.0)	2.00 (1.43, 2.81)*
Acute lower respiratory infection					
Males	27.6 (95.9)	11.5 (43.2)	1.82 (1.53, 2.15)*	29.4 (99.0)	1.02 (0.93, 1.11)
Females	28.4 (97.4)	10.0 (39.7)	2.33 (1.88, 2.89)*	29.4 (99.0)	1.01 (0.95, 1.06)
Chronic lower respiratory diseases					
Males	29.4 (99.0)	11.7 (43.7)	1.48 (1.21, 1.81)*	NA	
Females	28.7 (98.1)	-9.0 (1.0)	1.70 (1.28, 2.26)*	29.4 (99.0)	1.00 (0.97, 1.04)
COPD					
Males	29.4 (99.0)	-9.0 (1.0)	1.58 (1.02, 2.44)*	NA	
Females	21.6 (70.7)	-9.0 (1.0)	1.57 (0.80, 3.07)	29.4 (99.0)	1.56 (0.88, 2.77)
Asthma					
Males	-6.4 (3.4)	-9.0 (1.0)	1.02 (0.91, 1.14)	14.5 (50.2)	1.95 (1.51, 2.51)*
Females	29.4 (99.0)	8.9 (37.9)	2.42 (1.77, 3.31)*	NA	
ARDS and pulmonary oedema					
Males	23.3 (77.8)	-9.0 (1.0)	2.92 (1.20, 7.12)*	29.4 (99.0)	2.17 (1.12, 4.21)*
Females	29.4 (99.0)	-1.9 (11.1)	1.44 (0.57, 3.65)	NA	
ILD					
Males	-9.0 (1.0)	NA		3.6 (25.2)	1.45 (0.77, 2.72)
Females	25.1 (87.1)	-9.0 (1.0)	2.03 (0.80, 5.11)	29.4 (99.0)	1.09 (0.62, 1.90)
Pneumothorax					
Males	15.1 (51.9)	-3.8 (7.1)	1.18 (1.00, 1.40)*	29.4 (99.0)	1.17 (0.93, 1.48)
Females	7.5 (35.0)	-9.0 (1.0)	1.44 (0.80, 2.58)	29.4 (99.0)	1.58 (0.87, 2.88)

ED, emergency department; RR, relative risk; CI, confidence interval; MinRT, minimum risk temperature; MaxRT, maximum risk temperature; COPD, chronic obstructive pulmonary disease; ARDS, acute respiratory distress syndrome; ILD, interstitial lung disease; NA, not available

*p < 0.05

Regarding hot temperatures, both sexes showed an increased risk for total respiratory disease ED visits at MaxRT 29.4 °C (99.0%), with males (RR = 1.27, 95% CI = 1.12–1.43) and females (RR = 1.24, 95% CI = 1.08–1.42) having similar risk levels. This trend was also observed for AURI and influenza and pneumonia. However, males exhibited a higher risk for asthma and ARDS and pulmonary oedema at hot temperatures.

### Sensitivity analysis

Our sensitivity analysis, which used maximum and minimum temperatures, showed minor variations in RRs, but overall, the results supported our main findings (Additional file 1: Table S2 and Additional file 2: Fig. S1-S2). Specifically, when using maximum temperature, a significant association was found between a MaxRT of 31 °C and increased ED visits for pneumothorax (RR = 1.18, 95% CI = 1.00–1.40). In contrast, during hot weather, ARDS and pulmonary oedema were associated with increased ED visits when analysing minimum temperature (RR = 1.66, 95% CI = 1.03–2.69).

Our analysis examined the risk ratios for ED visits at various lag periods (7-day, 14-day, and 28-day) under both cold and hot conditions (Additional file 1: Table S3 and Additional file 2: Fig. S3-S5). Both heat and cold exposure demonstrated stronger effects with longer lags. Additionally, we observed that hot temperatures showed similar effects at both shorter and longer lags, whereas cold temperatures exhibited a stronger effect at longer lags compared to shorter lags. Sensitivity analyses, which included modifications to the DLNM model (Additional file 1: Table S4) and consideration of air pollution (Additional file 2: Fig. S6-S11), were consistent with our main findings.

## Discussion

This is the first comprehensive analysis of the association between ambient temperature and ED visits for various respiratory diseases in Seoul, South Korea. We found that both cold and hot temperatures significantly affected the frequency of ED visits for respiratory diseases, with the intensity of the effect varying across diseases and demographic groups. Cold temperatures were associated with an increased risk of ED visits for respiratory diseases overall, whereas hot temperatures were significantly associated with increased ED visits for AURI, influenza, and pneumonia. Cold weather increased ED visits for all ages, most in 18–64 group; hot weather mainly affected those under 18.

Our findings of increased ED visits for several respiratory diseases during colder temperatures are consistent with prior studies [11, 13, 15–18]. Chen et al. revealed that in Beijing, from 2012 to 2015, extreme cold (-6.5 °C) was associated with increased asthma hospitalisations (Cumulative RR = 2.3, 95% CI = 1.57–3.42) compared to the reference temperature (22 °C) [13]. Tseng et al. found that a 5 °C decrease in mean temperature over 28 days increased the long-term risk of COPD exacerbation (odds ratio [OR] = 1.106, 95% CI = 1.063–1.152) in Taiwan [15]. Similarly, a Chinese study demonstrated that both moderate (3 °C, OR = 1.68, 95% CI = 1.18–2.39) and extreme (-7 °C, OR = 1.73, 95% CI–1.13-2.67) cold temperatures were associated with a significant increase in the risk of asthma exacerbation [16]. The increased incidence of acute exacerbations of airway diseases during the cold season can be attributed to several factors. Breathing cold air tends to heighten bronchial reactivity, making the airways more sensitive and prone to inflammation and constriction [26]. Moreover, cold temperatures, particularly during the winter, are associated with an increase in respiratory infections, including pneumonia [17, 18, 27, 28]. Impairment of mucociliary clearance function at cold temperatures facilitates the onset of these infections [29, 30]. A study in Hong Kong revealed that temperature-related pneumonia hospitalisations accounted for 10.7% of cases, with 8.7% attributed to cold temperatures and 2.0% to hot temperatures [17]. Furthermore, low air temperatures are correlated with an increased incidence of influenza [28]. Our study corroborates these findings and highlights the significant role of cold temperatures in exacerbating respiratory diseases.

Our findings showed variations in MaxRT during the cold season for different respiratory diseases. Specifically, AURI, influenza, and COPD peaked at -9.0 °C, while asthma and ALRI showed increased ED visits at temperatures above 10 °C. The association between AURI and cold air temperatures may be due to the chilling effect on the nasal and upper respiratory tracts, which can worsen symptoms by impairing respiratory defences [27]. Consequently, the subzero MaxRT for AURI, a major trigger for COPD exacerbation, may explain the increased ED visits, especially among patients with COPD [31]. Interestingly, the peak temperatures for asthma and ALRI in our study were aligned with the average temperatures during the spring and autumn transitional seasons in South Korea. This finding was consistent with the study by Sohn et al., which demonstrated that each 1 °C increase in temperature above 6 °C, up to 14 °C, was associated with an additional risk of pneumonia of 1.89 (95% CI, 1.37–2.61) [19]. This suggested that significant daily temperature fluctuations during these seasons could potentially trigger the onset of symptoms associated with these diseases [32, 33]. This is further supported by a study by Kim et al., which revealed that a 1-unit increase in day-to-day temperature change led to a 3.5% increase in asthma-related ED visits, particularly in the fall and spring [20].

Conversely, our study identified a MinRT of 14 °C for pneumothorax, with a peak at -5.2 °C. The increase in pneumothorax cases during cold weather could be associated with swift alterations in temperature, atmospheric pressure, or humidity [34, 35], which might induce a rupture, culminating in pneumothorax. However, the exact relationship between temperature and the risk of pneumothorax requires further investigation.

We observed a significant increase in ED visits for respiratory diseases among individuals aged < 18 years, particularly during extremely hot temperatures. The vulnerability of different age groups to the effects of high temperatures on respiratory diseases remains debatable [10, 12–14, 21, 22]. Elderly individuals are often considered more susceptible because of physiological factors, pre-existing health conditions, and economic constraints [36]. This viewpoint is supported by the findings of a Brazilian cohort study on COPD and asthma hospitalizations [21, 22]. However, these studies have reported conflicting findings. A case crossover study conducted from 2007 to 2018 in England found that summer temperature increases significantly escalated asthma-related hospital admissions in individuals aged 16–64 years, but not in those under 16 or over 65 [10]. Similarly, the age group 65–74 showed the most pronounced increase in COPD hospital admissions due to heat effects compared to other age groups [12]. However, heat exposure may be more impactful in younger populations as they tend to be more active outdoors at higher temperatures. A study conducted in the USA reported that extreme summer heat events increased asthma hospitalisations the most among youths and adults, while extreme precipitation events affected those aged 4 years or younger the most [14]. Similarly, a Chinese study demonstrated that the risk of asthma hospitalisation due to hot temperatures was higher in younger individuals (19–64 years old) than in those aged > 65 years [13]. A recent meta-analysis revealed that cold temperatures were associated with asthma exacerbations in children (< 19 years) and the elderly (≥ 65 years) but not in adults (16–64 years), while hot temperatures were associated with increased asthma exacerbations in all age groups, with the highest risk in adults [37]. Geographical, climatic, and population variations may explain these discrepancies. In addition, our study focused on ED visits rather than total hospital admissions, accounting for various influencing factors such as healthcare facility availability and accessibility and individual health-seeking behaviours.

This study had several limitations. First, ambient temperature was used as a proxy for personal exposure, which may not perfectly represent individual exposure levels. Although exposure measurement errors may have led to an underestimation of the results, the findings remain noteworthy. Second, the study relied on ED visits as the primary outcome, which may not have captured all instances of respiratory disease exacerbation. However, ED visits are critical indicators of severe disease exacerbation and remain in this context. Third, the study was conducted in Seoul, South Korea; therefore, the findings may not be generalisable to other regions with different climates, health behaviours, and healthcare systems. Finally, the study did not account for potential confounding factors such as indoor air quality, individual health behaviours, and pre-existing health conditions. Nevertheless, the case-crossover design inherently controlled for individual characteristics that did not vary over a brief period. Despite these limitations, our study stands out due to its extensive dataset, unique focus on MaxRT for various respiratory diseases, advanced statistical methods, and significant public health implications.

## Conclusion

In conclusion, our study highlights the significant influence of ambient temperature on the frequency of ED visits for various respiratory diseases. This underscores the importance of considering weather patterns in developing public health strategies for managing respiratory diseases.

### Electronic supplementary material

Below is the link to the electronic supplementary material.

**Additional file 1**. **Table S1**. Quasi-Akaike information criteria (QAIC) values for the association between ambient mean temperature and emergency department visits for respiratory diseases according to various distributed lag nonlinear model specifications; **Table S2**. Sensitivity analyses employing maximum and minimum temperature as temperature measures; **Table S3**. Sensitivity analyses altering lag structures for distributed lag nonlinear models; **Table S4**. Sensitivity analyses altering model specifications of exposure functions in distributed lag nonlinear models for results of total respiratory diseases.

**Additional file 2**. **Fig. S1**. Sensitivity analysis conducted for the association between maximum ambient temperature and emergency department visits for various subtypes of respiratory diseases; **Fig. 2**. Sensitivity analysis conducted for the association between minimum ambient temperature and emergency department visits for various subtypes of respiratory diseases; **Fig. S3**. Sensitivity analysis of mean ambient temperature and emergency department visits for various respiratory disease subtypes over 0–7 lag days.; **Fig. S4**. Sensitivity analysis of mean ambient temperature and emergency department visits for various respiratory disease subtypes over 0–14 lag days; **Fig. S5** Sensitivity analysis of mean ambient temperature and emergency department visits for various respiratory disease subtypes over 0–28 lag days; **Fig. S6**. Sensitivity analysis of the association between mean ambient temperature and emergency department visits for various subtypes of respiratory diseases, adjusted for PM10 concentration; **Fig. S7**. Sensitivity analysis of the association between mean ambient temperature and emergency department visits for various subtypes of respiratory diseases, adjusted for PM2.5 concentration; **Fig. S8**. Sensitivity analysis of the association between mean ambient temperature and emergency department visits for various subtypes of respiratory diseases, adjusted for NO2 concentration; **Fig. S9**. Sensitivity analysis of the association between mean ambient temperature and emergency department visits for various subtypes of respiratory diseases, adjusted for O3 concentration; **Fig. S10**. Sensitivity analysis of the association between mean ambient temperature and emergency department visits for various subtypes of respiratory diseases, adjusted for SO2 concentration; **Fig. S11**. Sensitivity analysis of the association between mean ambient temperature and emergency department visits for various subtypes of respiratory diseases, adjusted for CO concentration.

## Data Availability

The data supporting this study’s findings are available from the corresponding author upon reasonable request.

## Abbreviations

COPD: Chronic obstructive pulmonary disease
CI: Confidence interval
ALRI: Acute lower respiratory infection
CLRD: Chronic lower respiratory disease
ED: Emergency department
AURI: Acute upper respiratory infection
ILD: Interstitial lung disease
RR: Relative risk
DLNM: Distributed lag nonlinear model
MaxRT: Maximum risk temperature
MinRT: Minimum risk temperature
NEDIS: National emergency department information system
