# Supplementary material for: Impact of ambient temperature on respiratory disease: a case-crossover study in Seoul
Source: Respir Res. 2024 Feb 5;25:73. doi: 10.1186/s12931-024-02699-0 (PMC10845516; doi:10.1186/s12931-024-02699-0)
Supplement: Supplementary file 1 — Additional file 1. Table S1. Quasi-Akaike information criteria (QAIC) values for the association between ambient mean temperature and emergency department visits for respiratory diseases according to various distributed lag nonlinear model specifications; Table S2. Sensitivity analyses employing maximum and minimum temperature as temperature measures; Table S3. Sensitivity analyses altering lag structures for distributed lag nonlinear models; Table S4. Sensitivity analyses altering model specifications of exposure functions in distributed lag nonlinear models for results of total respiratory diseases. [file 12931_2024_2699_MOESM1_ESM.docx]

**Impact of ambient temperature on respiratory disease: a case-crossover study in Seoul**

Hyewon Lee^1,2^, Hee-Young Yoon^3^

^1^Department of Health Administration and Management, College of Medical Sciences, Soonchunhyang University, Asan, Republic of Korea

^2^Department of Software Convergence, Soonchunhyang University Graduate School, Asan, Republic of Korea

^3^Division of Allergy and Respiratory Diseases, Department of Internal Medicine, Soonchunhyang University Seoul Hospital, Seoul, Republic of Korea

**Table S1.** Quasi-Akaike information criteria (QAIC) values for the association between ambient mean temperature and emergency department visits for respiratory diseases according to various distributed lag nonlinear model specifications.

|  | QAIC |
| --- | --- |
| 1 knot at 50th percentile | 97180.3 |
| 2 knots at |  |
| equally spaced places | 94029.2 |
| 33.3rd and 66.7th | 93344.9* |
| 25th and 75th | 94092.5 |
| 10th and 90th | 94567.9 |
| 3 knots at |  |
| 25th, 50th, and 75th | 94118.6 |
| 10th, 75th, and 90th | 93361.3 |
| 10th, 50th, and 90th | 94561.7 |
| Cubic B-spine | 97002.4 |

*Main model

**Table S2.** Sensitivity analyses employing maximum and minimum temperature as temperature measures.

| Temperature measure | MinRT (%) | Cold temperature | | Hot temperature | |
| --- | --- | --- | --- | --- | --- |
|  |  | MaxRT (%) | RR (95% CI) | MaxRT (%) | RR (95% CI) |
| Maximum temperature (–5.5℃ to 33.9℃) ^a^ | |  |  |  |  |
| Total respiratory diseases | 28.9 (84.2) | -5.5 (1.0) | 2.78 (2.35, 3.30)* | 33.9 (99.0) | 1.40 (1.21, 1.63)* |
| Acute upper respiratory infection | 28.5 (82.1) | -5.5 (1.0) | 2.06 (1.72, 2.47)* | 33.9 (99.0) | 1.33 (1.14, 1.56)* |
| Influenza and pneumonia | 29.0 (84.8) | -5.5 (1.0) | 12.39 (9.72, 15.8)* | 33.9 (99.0) | 2.09 (1.60, 2.73)* |
| Acute lower respiratory infection | 31.0 (93.6) | 15.5 (42.0) | 1.95 (1.70, 2.23)* | 33.9 (99.0) | 1.06 (0.93, 1.20) |
| Chronic lower respiratory diseases | 33.7 (98.8) | 14.6 (40.0) | 1.49 (1.26, 1.76)* | 33.9 (99.0) | 1.00 (0.99, 1.01) |
| COPD | 32.2 (96.8) | -5.5 (1.0) | 1.51 (1.05, 2.18)* | 33.9 (99.0) | 1.00 (0.90, 1.12) |
| Asthma | 33.9 (99.0) | 17.1 (45.2) | 1.78 (1.39, 2.29)* | NA | |
| ARDS and pulmonary oedema | 30.9 (93.2) | -4.0 (2.2) | 1.94 (1.00, 3.75)* | 33.9 (99.0) | 1.04 (0.72, 1.49) |
| ILD | 33.5 (98.6) | 10.0 (31.0) | 1.29 (0.75, 2.25) | 33.9 (99.0) | 1.00 (0.95, 1.05) |
| Pneumothorax | 17.5 (46.1) | -0.8 (5.8) | 1.19 (1.02, 1.39)* | 31.0 (93.6) | 1.18 (1.00, 1.40)* |
| Minimum temperature (–12.5℃ to 26.4℃) ^a^ | |  |  |  |  |
| Total respiratory diseases | 21.2 (84.5) | -12.5 (1.0) | 2.79 (2.36, 3.29)* | 26.4 (99.0) | 1.27 (1.13, 1.42)* |
| Acute upper respiratory infection | 20.7 (82.0) | -12.5 (1.0) | 2.14 (1.79, 2.55)* | 26.4 (99.0) | 1.24 (1.10, 1.40)* |
| Influenza and pneumonia | 21.5 (85.6) | -12.5 (1.0) | 11.55 (9.10, 14.66)* | 26.4 (99.0) | 1.63 (1.32, 2.01)* |
| Acute lower respiratory infection | 25.2 (97.0) | 10.0 (49.4) | 1.95 (1.68, 2.27)* | 26.4 (99.0) | 1.01 (0.96, 1.05) |
| Chronic lower respiratory diseases | 25.4 (97.3) | 6.3 (41.2) | 1.51 (1.31, 1.73)* | 26.4 (99.0) | 1.00 (0.97, 1.03) |
| COPD | 19.7 (77.9) | -12.5 (1.0) | 1.55 (1.13, 2.13)* | 26.4 (99.0) | 1.07 (0.85, 1.34) |
| Asthma | 26.4 (99.0) | 10.0 (49.4) | 1.89 (1.52, 2.35)* | NA | |
| ARDS and pulmonary oedema | 19.3 (76.7) | -7.3 (7.8) | 1.87 (1.14, 3.09)* | 26.4 (99.0) | 1.66 (1.03, 2.69)* |
| ILD | 22.2 (88.1) | -12.5 (1.0) | 1.35 (0.73, 2.50) | 26.4 (99.0) | 1.05 (0.77, 1.43) |
| Pneumothorax | 10.8 (52.1) | -12.5 (1.0) | 1.19 (0.95, 1.49) | 26.4 (99.0) | 1.21 (0.98, 1.48) |

RR, relative risk; CI, confidence interval; COPD, chronic obstructive pulmonary disease; ARDS, acute respiratory distress syndrome; MinRT, minimum risk temperature; MaxRT, maximum risk temperature; ILD, interstitial lung disease; RR, relative risk; NA, not available.

*p<0.05

**Table S3.** Sensitivity analyses altering lag structures for distributed lag nonlinear models.

| Model specifications | MinRT (°C) | Cold temperature | | Hot temperature | |
| --- | --- | --- | --- | --- | --- |
|  |  | MaxRT (°C) | RR (95% CI) | MaxRT (%) | RR (95% CI) |
| *7-day lag* |  |  |  |  |  |
| Total respiratory diseases | 24.7 | -9.0 | 1.20 (1.04, 1.38)* | 29.4 | 1.12 (1.02, 1.23)* |
|  |  |  |  |  |  |
| Acute upper respiratory infection | -1.1 | -9.0 | 1.08 (0.99, 1.18) | 29.4 | 1.12 (0.96, 1.3) |
| Influenza and pneumonia | 25.0 | -9.0 | 2.10 (1.70, 2.59)* | 29.4 | 1.30 (1.08, 1.56)* |
| Acute lower respiratory infection | 29.4 | 10.3 | 1.39 (1.18, 1.63)* | NA | |
| Chronic lower respiratory diseases | 29.4 | 12.1 | 1.29 (1.14, 1.46)* | NA | |
| COPD | 22.8 | 10.0 | 1.11 (0.94, 1.30) | 29.4 | 1.15 (0.96, 1.37) |
| Asthma | 29.4 | 13.9 | 1.55 (1.29, 1.86)* | NA | |
| ARDS and pulmonary oedema | 26.4 | -2.7 | 1.54 (0.97, 2.45) | 29.4 | 1.03 (0.82, 1.29) |
| ILD | 25.2 | 2.9 | 1.29 (0.90, 1.86) | 29.4 | 1.05 (0.82, 1.35) |
| Pneumothorax | 12.7 | -0.7 | 1.14 (1.03, 1.27)* | 27.5 | 1.21 (1.05, 1.39)* |
| *14-day lag* |  |  |  |  |  |
| Total respiratory diseases | 24.9 | -9.0 | 1.61 (1.38, 1.88)* | 29.4 | 1.18 (1.05, 1.32)* |
| Acute upper respiratory infection | 24.2 | -9.0 | 1.34 (1.14, 1.57)* | 29.4 | 1.18 (1.05, 1.32)* |
| Influenza and pneumonia | 25.3 | -9.0 | 3.92 (3.13, 4.92)* | 29.4 | 1.42 (1.16, 1.74)* |
| Acute lower respiratory infection | 29.4 | 10.3 | 1.80 (1.49, 2.16)* | NA | |
| Chronic lower respiratory diseases | 29.4 | 10.6 | 1.51 (1.29, 1.75)* | NA | |
| COPD | 23.4 | -1.0 | 1.28 (1.03, 1.60)* | 29.4 | 1.12 (0.90, 1.38) |
| Asthma | 29.4 | 13.1 | 1.90 (1.53, 2.36)* | NA | |
| ARDS and pulmonary oedema | 29.4 | -9.0 | 2.72 (1.31, 5.67)* | NA | |
| ILD | 29.4 | -5.6 | 1.55 (0.87, 2.77) | NA | |
| Pneumothorax | 12.1 | -9.0 | 1.14 (0.95, 1.38) | 29.4 | 1.23 (1.00, 1.51)* |
| *28-day lag* |  |  |  |  |  |
| Total respiratory diseases | 24.8 | -9.0 | 2.23 (1.84, 2.69)* | 29.4 | 1.25 (1.09, 1.43)* |
| Acute upper respiratory infection | 24.1 | -9.0 | 1.62 (1.33, 1.98)* | 29.4 | 1.25 (1.08, 1.45)* |
| Influenza and pneumonia | 25.2 | -9.0 | 8.99 (6.85, 11.81)* | 29.4 | 1.52 (1.20, 1.93)* |
| Acute lower respiratory infection | 26.9 | 10.4 | 2.07 (1.80, 2.38)* | 29.4 | 1.04 (0.94, 1.16) |
| Chronic lower respiratory diseases | 29.4 | 10.0 | 1.58 (1.33, 1.88)* | NA | |
| COPD | 26.0 | -9.0 | 1.73 (1.20, 2.51)* | 29.4 | 1.02 (0.84, 1.24) |
| Asthma | 29.4 | 12.8 | 2.07 (1.62, 2.65)* | NA | |
| ARDS and pulmonary oedema | 23.4 | -9.0 | 2.14 (1.00, 4.55)* | 29.4 | 1.54 (0.89, 2.68) |
| ILD | 29.4 | 10.0 | 1.49 (0.84, 2.61) | NA | |
| Pneumothorax | 14.1 | -1.8 | 1.17 (1.00, 1.36)* | 29.4 | 1.21 (0.96, 1.53) |

ED, emergency department; RR, relative risk; CI, confidence interval; MinRT, minimum risk temperature; MaxRT, maximum risk temperature; COPD, chronic obstructive pulmonary disease; ARDS, acute respiratory distress syndrome; ILD, interstitial lung disease; NA, not available.

*p<0.05

**Table S4.** Sensitivity analyses altering model specifications of exposure functions in distributed lag nonlinear models for results of total respiratory diseases.

| Model specifications | MinRT (°C) | Cold temperature | | Hot temperature | |
| --- | --- | --- | --- | --- | --- |
|  |  | MaxRT (°C) | RR (95% CI) | MaxRT (%) | RR (95% CI) |
| Main model (33.3rd and 63.7th) | 24.8 | –9.0 | 2.68 (2.26, 3.17)* | 29.4 | 1.26 (1.11, 1.42)* |
| 1 knot at 50th percentile | 29.4 | –9.0 | 2.27 (1.84, 2.80)* | NA | |
| 2 knots at |  |  |  |  |  |
| equally spaced places | 26.4 | –9.0 | 2.76 (2.30, 3.31)* | 29.4 | 1.03 (0.96, 1.10) |
| 25th and 75th | 25.5 | –9.0 | 2.86 (2.39, 3.41)* | 29.4 | 1.25 (1.09, 1.43)* |
| 10th and 90th | 26.8 | –9.0 | 3.03 (2.52, 3.65)* | 29.4 | 1.23 (1.06, 1.44)* |
| 3 knots at |  |  |  |  |  |
| 25th, 50th, and 75th | 25.5 | –9.0 | 2.83 (2.37, 3.38)* | 29.4 | 1.20 (1.04, 1.38)* |
| 10th, 75th, and 90th | 29.4 | –9.0 | 2.73 (2.16, 3.46)* | NA | |
| 10th, 50th, and 90th | 26.8 | –9.0 | 2.93 (2.42, 3.54)* | 29.4 | 1.17 (0.99, 1.38) |
| Cubic B-spine | 29.4 | –9.0 | 2.43 (1.97, 2.99)* | NA | |

RR, relative risk; CI, confidence interval; MinRT, minimum risk temperature; MaxRT, maximum risk temperature; RR, relative risk; NA, not available.

*p<0.05
