# Supplementary material for: Impact of ambient temperature on respiratory disease: a case-crossover study in Seoul
Source: Respir Res. 2024 Feb 5;25:73. doi: 10.1186/s12931-024-02699-0 (PMC10845516; doi:10.1186/s12931-024-02699-0)
Supplement: Supplementary file 2 — Additional file 2. Fig. S1. Sensitivity analysis conducted for the association between maximum ambient temperature and emergency department visits for various subtypes of respiratory diseases; Fig. 2. Sensitivity analysis conducted for the association between minimum ambient temperature and emergency department visits for various subtypes of respiratory diseases; Fig. S3. Sensitivity analysis of mean ambient temperature and emergency department visits for various respiratory disease subtypes over 0–7 lag days.; Fig. S4. Sensitivity analysis of mean ambient temperature and emergency department visits for various respiratory disease subtypes over 0–14 lag days; Fig. S5 Sensitivity analysis of mean ambient temperature and emergency department visits for various respiratory disease subtypes over 0–28 lag days; Fig. S6. Sensitivity analysis of the association between mean ambient temperature and emergency department visits for various subtypes of respiratory diseases, adjusted for PM10 concentration; Fig. S7. Sensitivity analysis of the association between mean ambient temperature and emergency department visits for various subtypes of respiratory diseases, adjusted for PM2.5 concentration; Fig. S8. Sensitivity analysis of the association between mean ambient temperature and emergency department visits for various subtypes of respiratory diseases, adjusted for NO2 concentration; Fig. S9. Sensitivity analysis of the association between mean ambient temperature and emergency department visits for various subtypes of respiratory diseases, adjusted for O3 concentration; Fig. S10. Sensitivity analysis of the association between mean ambient temperature and emergency department visits for various subtypes of respiratory diseases, adjusted for SO2 concentration; Fig. S11. Sensitivity analysis of the association between mean ambient temperature and emergency department visits for various subtypes of respiratory diseases, adjusted for CO concentration. [file 12931_2024_2699_MOESM2_ESM.docx]

**Impact of ambient temperature on respiratory disease: a case-crossover study in Seoul**

Hyewon Lee^1,2^, Hee-Young Yoon^3^

^1^Department of Health Administration and Management, College of Medical Sciences, Soonchunhyang University, Asan, Republic of Korea

^2^Department of Software Convergence, Soonchunhyang University Graduate School, Asan, Republic of Korea

^3^Division of Allergy and Respiratory Diseases, Department of Internal Medicine, Soonchunhyang University Seoul Hospital, Seoul, Republic of Korea

**Fig. S1.** Sensitivity analysis conducted for the association between maximum ambient temperature and emergency department visits for various subtypes of respiratory diseases.


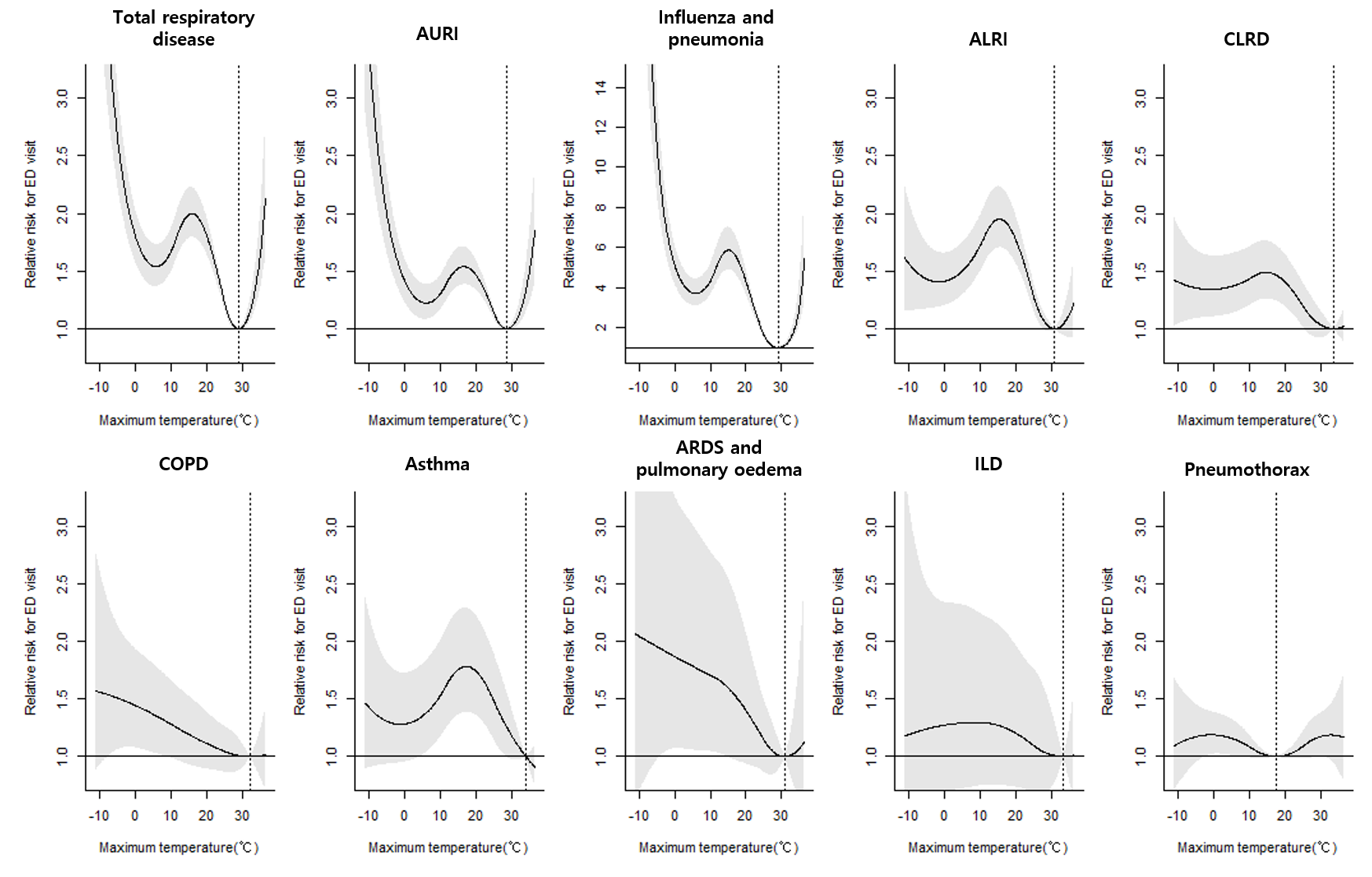


Dotted vertical lines indicate MinRT, while the solid line with shaded area represents relative risk with 95% confidence intervals.

ED, emergency department; MinRT, minimum risk temperature; AURI, acute upper respiratory infection; ALRI, acute lower respiratory infection; CLRD, chronic lower respiratory disease; COPD, chronic obstructive pulmonary disease; ARDS, acute respiratory distress syndrome; ILD, interstitial lung disease

**Fig. 2.** Sensitivity analysis conducted for the association between minimum ambient temperature and emergency department visits for various subtypes of respiratory diseases.


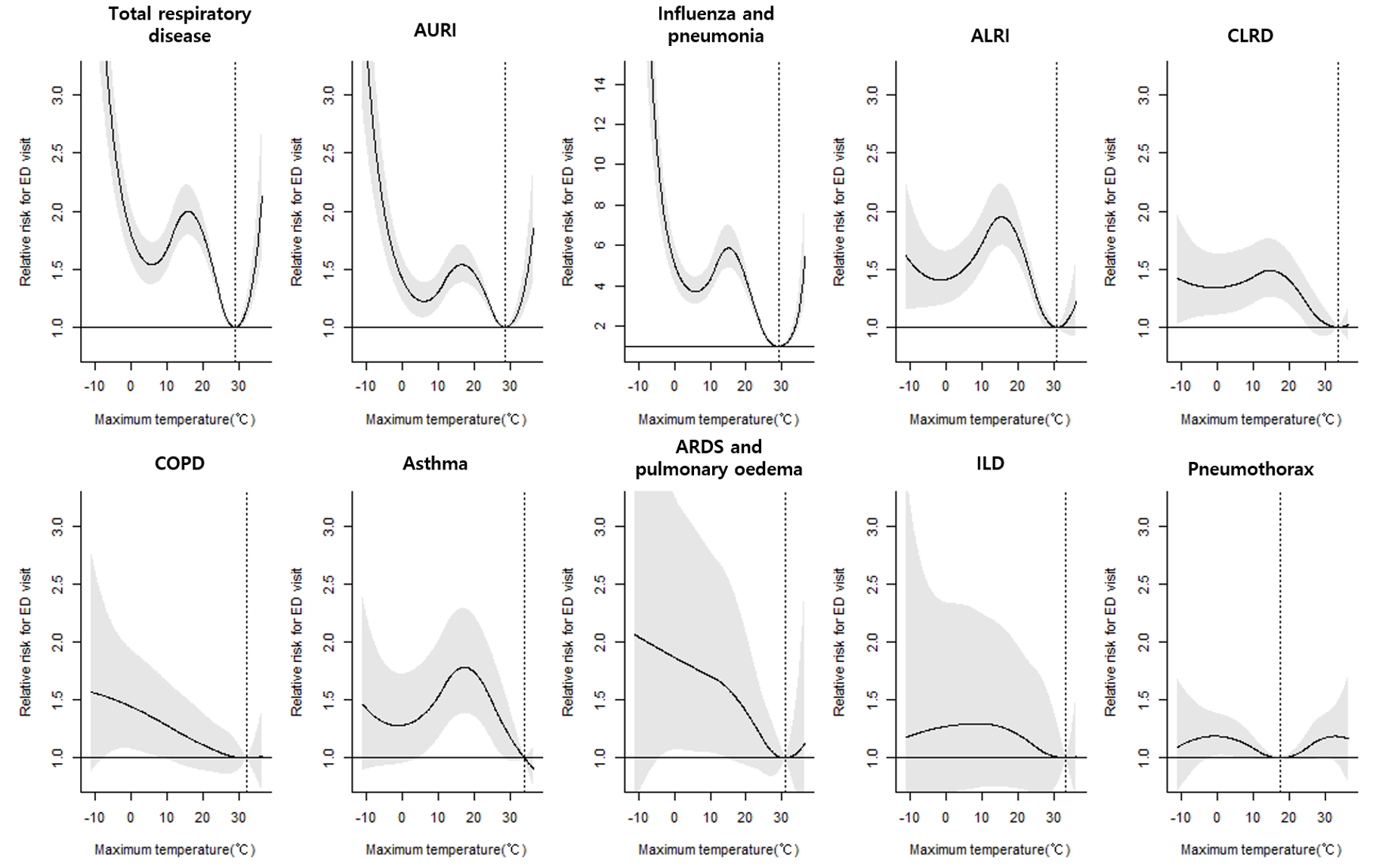


Dotted vertical lines indicate MinRT, while the solid line with shaded area represents relative risk with 95% confidence intervals.

ED, emergency department; MinRT, minimum risk temperature; AURI, acute upper respiratory infection; ALRI, acute lower respiratory infection; CLRD, chronic lower respiratory disease; COPD, chronic obstructive pulmonary disease; ARDS, acute respiratory distress syndrome; ILD, interstitial lung disease

**Fig. S3.** Sensitivity analysis of mean ambient temperature and emergency department visits for various respiratory disease subtypes over 0-7 lag days.


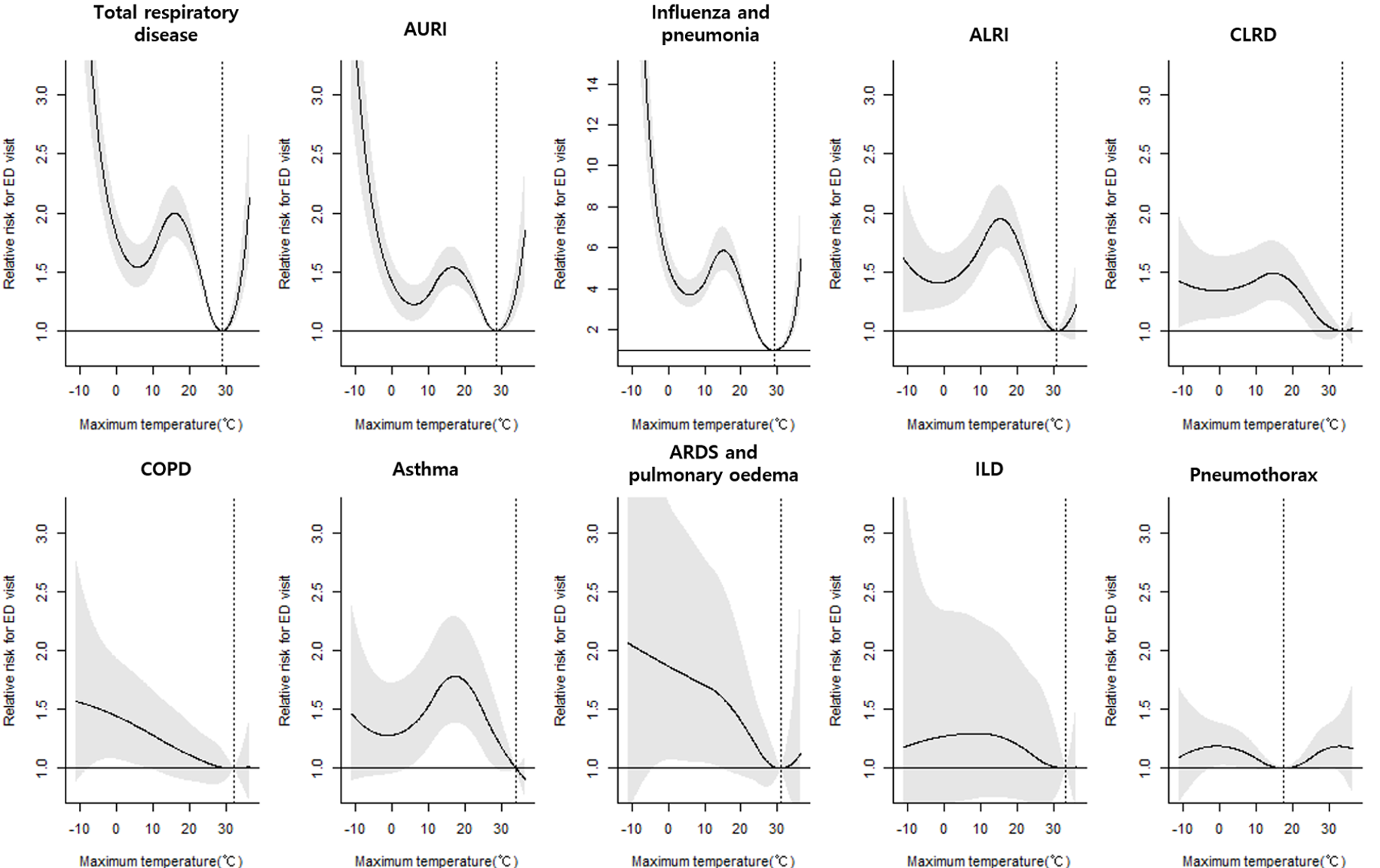


Dotted vertical lines indicate MinRT, while the solid line with shaded area represents relative risk with 95% confidence intervals.

ED, emergency department; MinRT, minimum risk temperature; AURI, acute upper respiratory infection; ALRI, acute lower respiratory infection; CLRD, chronic lower respiratory disease; COPD, chronic obstructive pulmonary disease; ARDS, acute respiratory distress syndrome; ILD, interstitial lung disease

**Fig. S4.** Sensitivity analysis of mean ambient temperature and emergency department visits for various respiratory disease subtypes over 0-14 lag days.


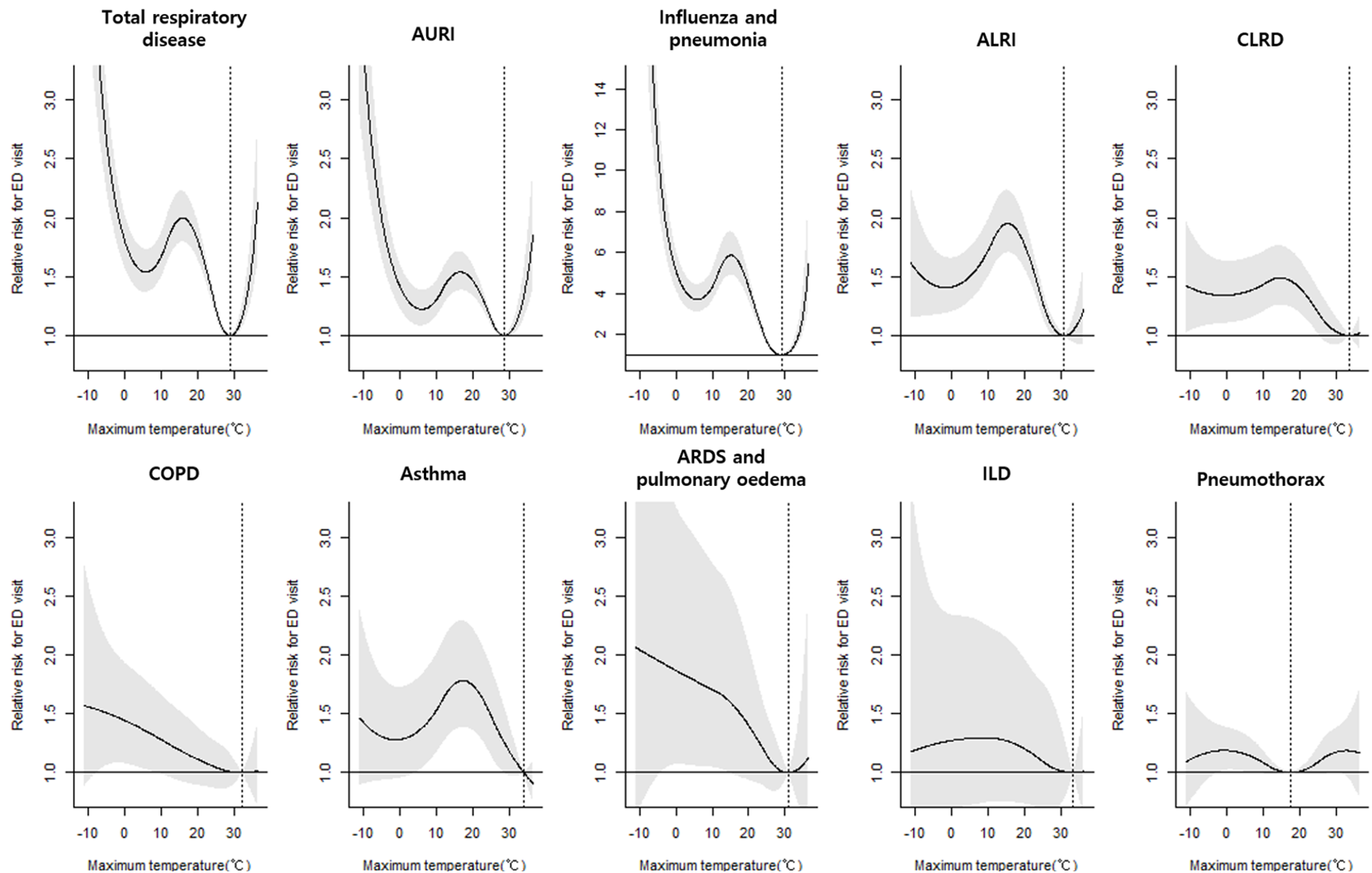


Dotted vertical lines indicate MinRT, while the solid line with shaded area represents relative risk with 95% confidence intervals.

ED, emergency department; MinRT, minimum risk temperature; AURI, acute upper respiratory infection; ALRI, acute lower respiratory infection; CLRD, chronic lower respiratory disease; COPD, chronic obstructive pulmonary disease; ARDS, acute respiratory distress syndrome; ILD, interstitial lung disease

**Fig. S5** Sensitivity analysis of mean ambient temperature and emergency department visits for various respiratory disease subtypes over 0-28 lag days.





Dotted vertical lines indicate MinRT, while the solid line with shaded area represents relative risk with 95% confidence intervals.

ED, emergency department; MinRT, minimum risk temperature; AURI, acute upper respiratory infection; ALRI, acute lower respiratory infection; CLRD, chronic lower respiratory disease; COPD, chronic obstructive pulmonary disease; ARDS, acute respiratory distress syndrome; ILD, interstitial lung disease

**Fig. S6.** Sensitivity analysis of the association between mean ambient temperature and emergency department visits for various subtypes of respiratory diseases, adjusted for PM_10_ concentration.


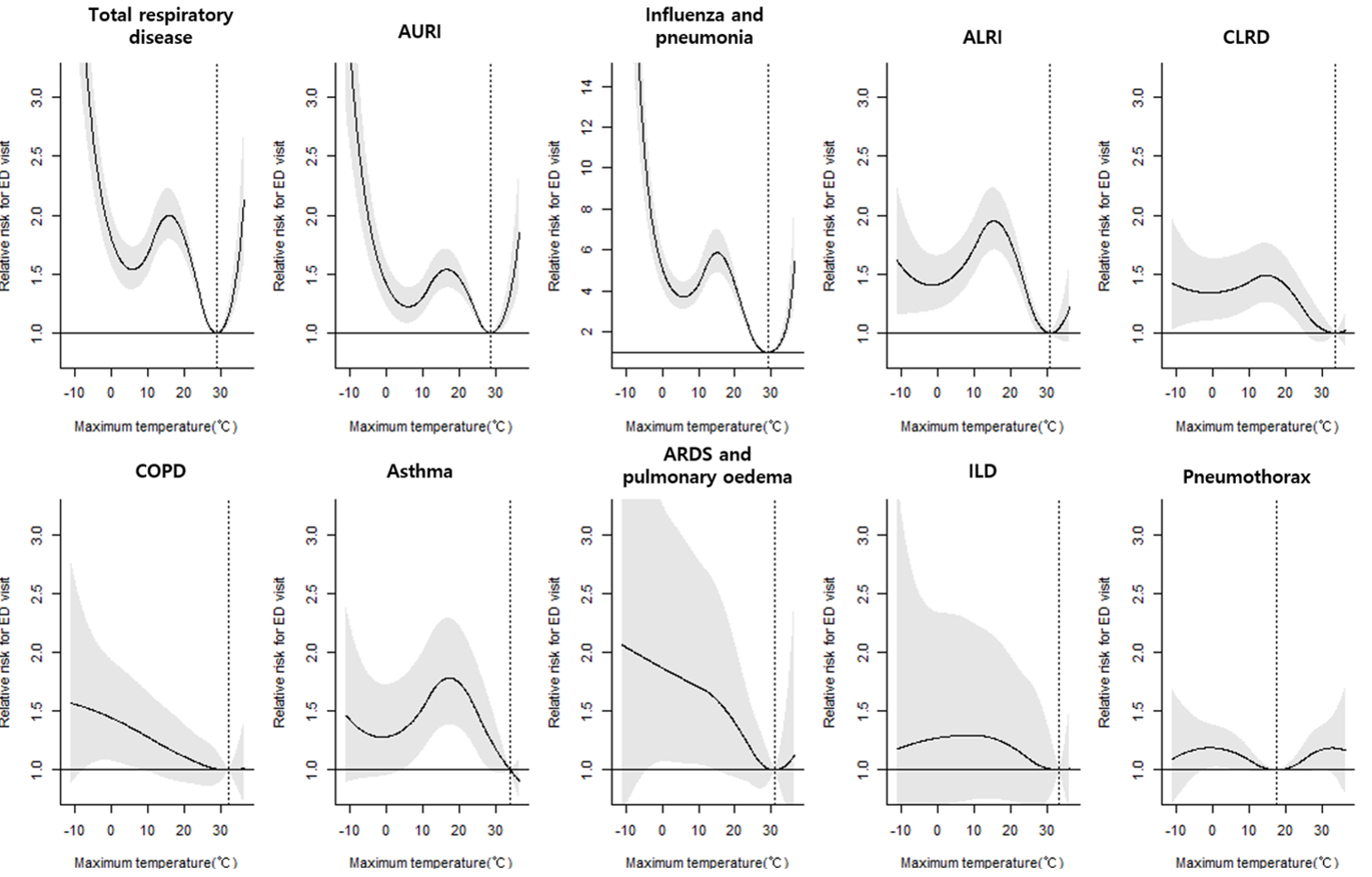


Dotted vertical lines indicate MinRT, while the solid line with shaded area represents relative risk with 95% confidence intervals.

PM_10_, particulate matter 10 or less in diameter; RR, relative risk; MinRT, minimum risk temperature; AURI, acute upper respiratory infection; ALRI, acute lower respiratory infection; CLRD, chronic lower respiratory disease; COPD, chronic obstructive pulmonary disease; ARDS, acute respiratory distress syndrome; ILD, interstitial lung disease

**Fig. S7.** Sensitivity analysis of the association between mean ambient temperature and emergency department visits for various subtypes of respiratory diseases, adjusted for PM_2.5_ concentration.


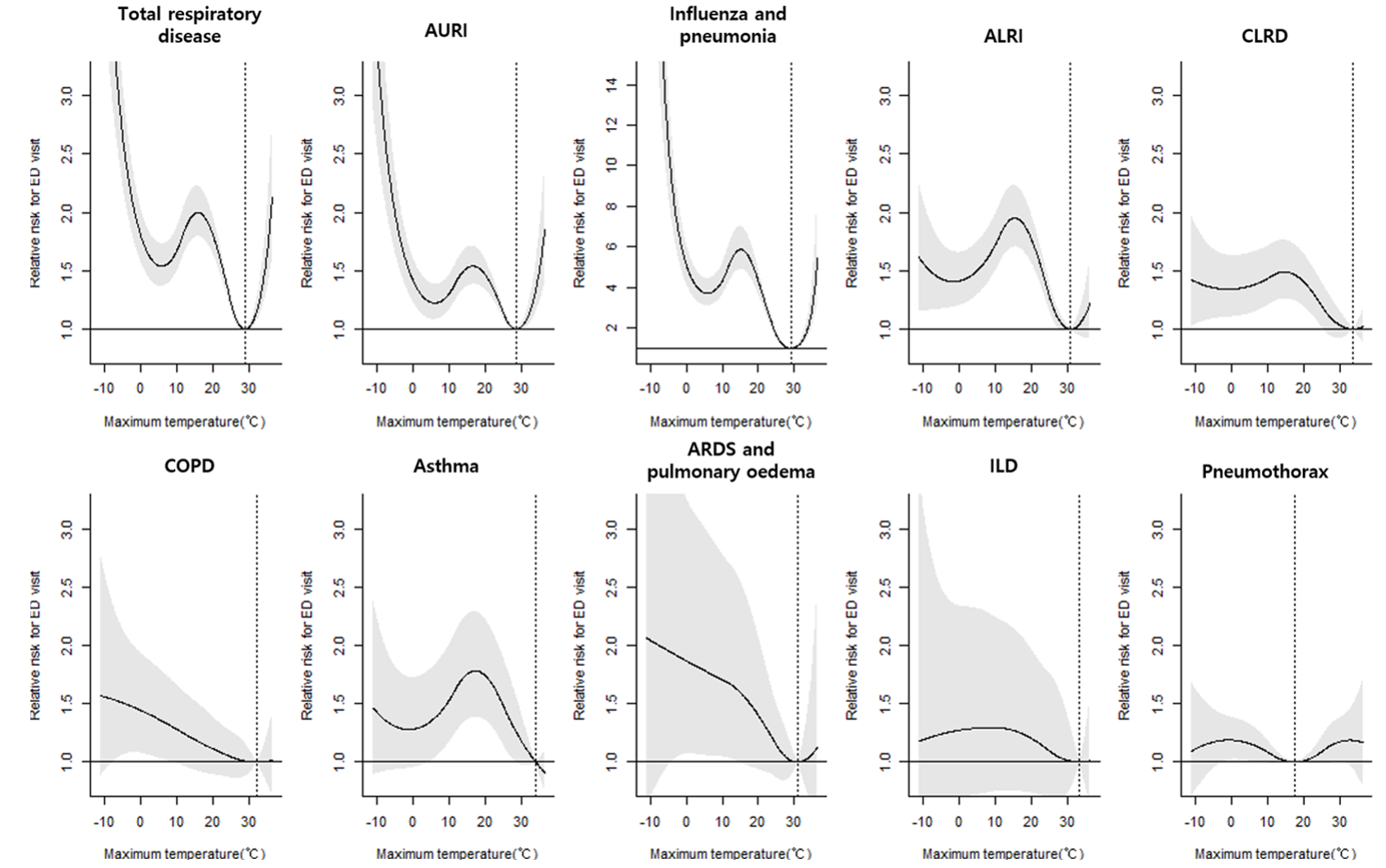


Dotted vertical lines indicate MinRT, while the solid line with shaded area represents relative risk with 95% confidence intervals.

PM_2.5_, particulate matter 2.5 or less in diameter; RR, relative risk; MinRT, minimum risk temperature; AURI, acute upper respiratory infection; ALRI, acute lower respiratory infection; CLRD, chronic lower respiratory disease; COPD, chronic obstructive pulmonary disease; ARDS, acute respiratory distress syndrome; ILD, interstitial lung disease

**Fig. S8.** Sensitivity analysis of the association between mean ambient temperature and emergency department visits for various subtypes of respiratory diseases, adjusted for NO_2_ concentration.


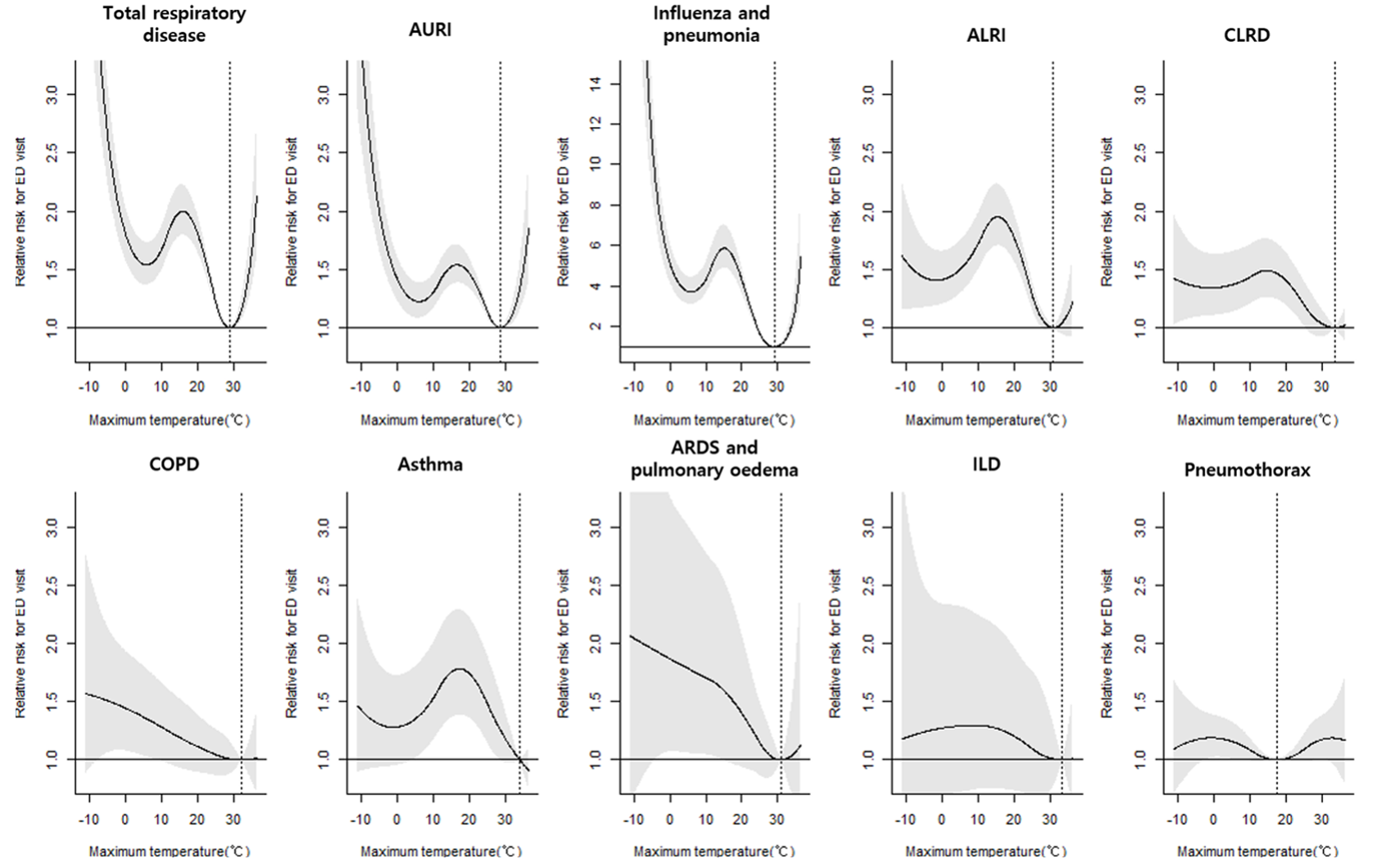


Dotted vertical lines indicate MinRT, while the solid line with shaded area represents relative risk with 95% confidence intervals.

NO_2_, nitrogen dioxide; RR, relative risk; MinRT, minimum risk temperature; AURI, acute upper respiratory infection; ALRI, acute lower respiratory infection; CLRD, chronic lower respiratory disease; COPD, chronic obstructive pulmonary disease; ARDS, acute respiratory distress syndrome; ILD, interstitial lung disease

**Fig. S9.** Sensitivity analysis of the association between mean ambient temperature and emergency department visits for various subtypes of respiratory diseases, adjusted for O_3_ concentration.


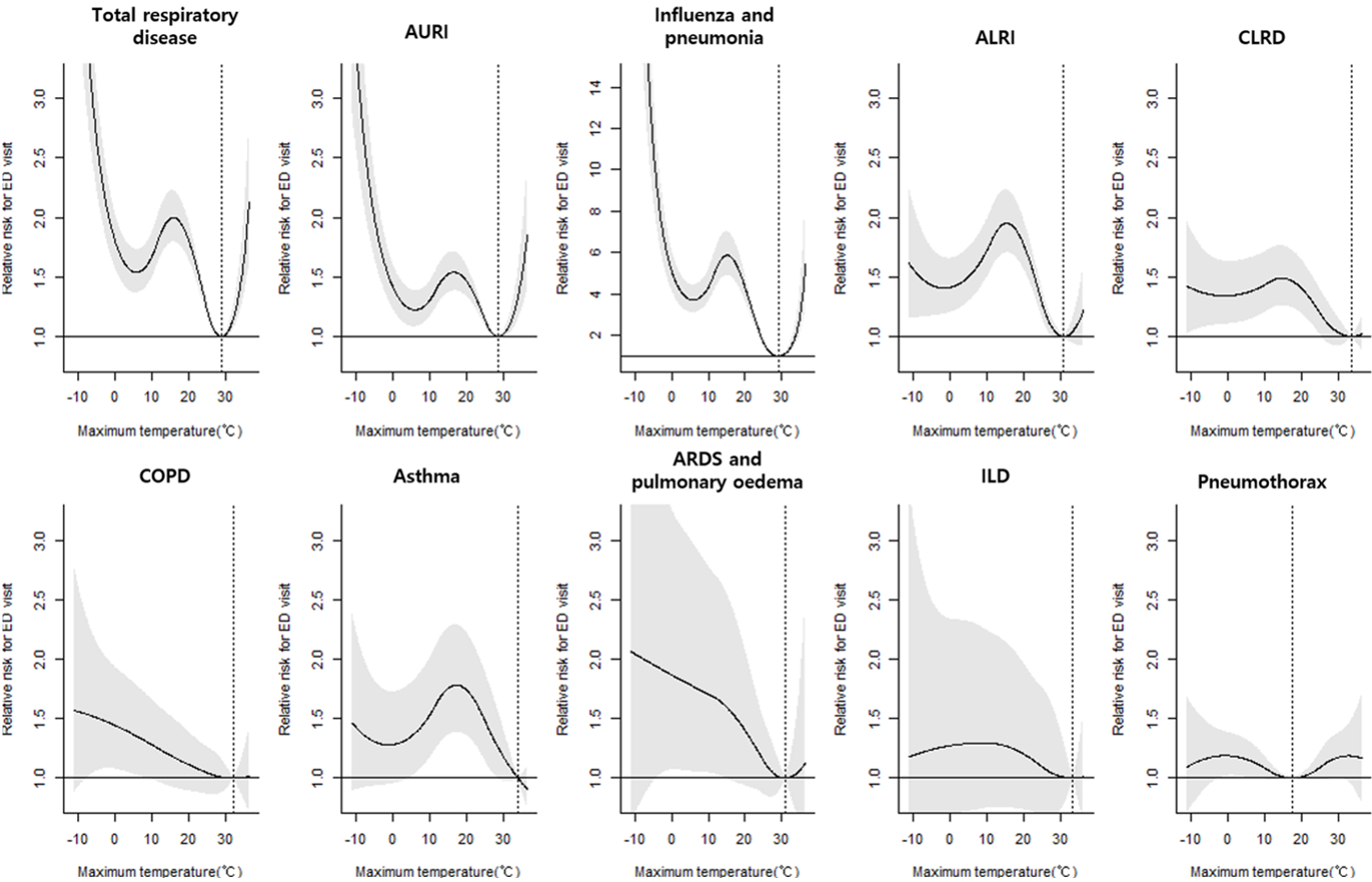


Dotted vertical lines indicate MinRT, while the solid line with shaded area represents relative risk with 95% confidence intervals.

O_3_, ozone; RR, relative risk; MinRT, minimum risk temperature; AURI, acute upper respiratory infection; ALRI, acute lower respiratory infection; CLRD, chronic lower respiratory disease; COPD, chronic obstructive pulmonary disease; ARDS, acute respiratory distress syndrome; ILD, interstitial lung disease

**Fig. S10.** Sensitivity analysis of the association between mean ambient temperature and emergency department visits for various subtypes of respiratory diseases, adjusted for SO_2_ concentration.


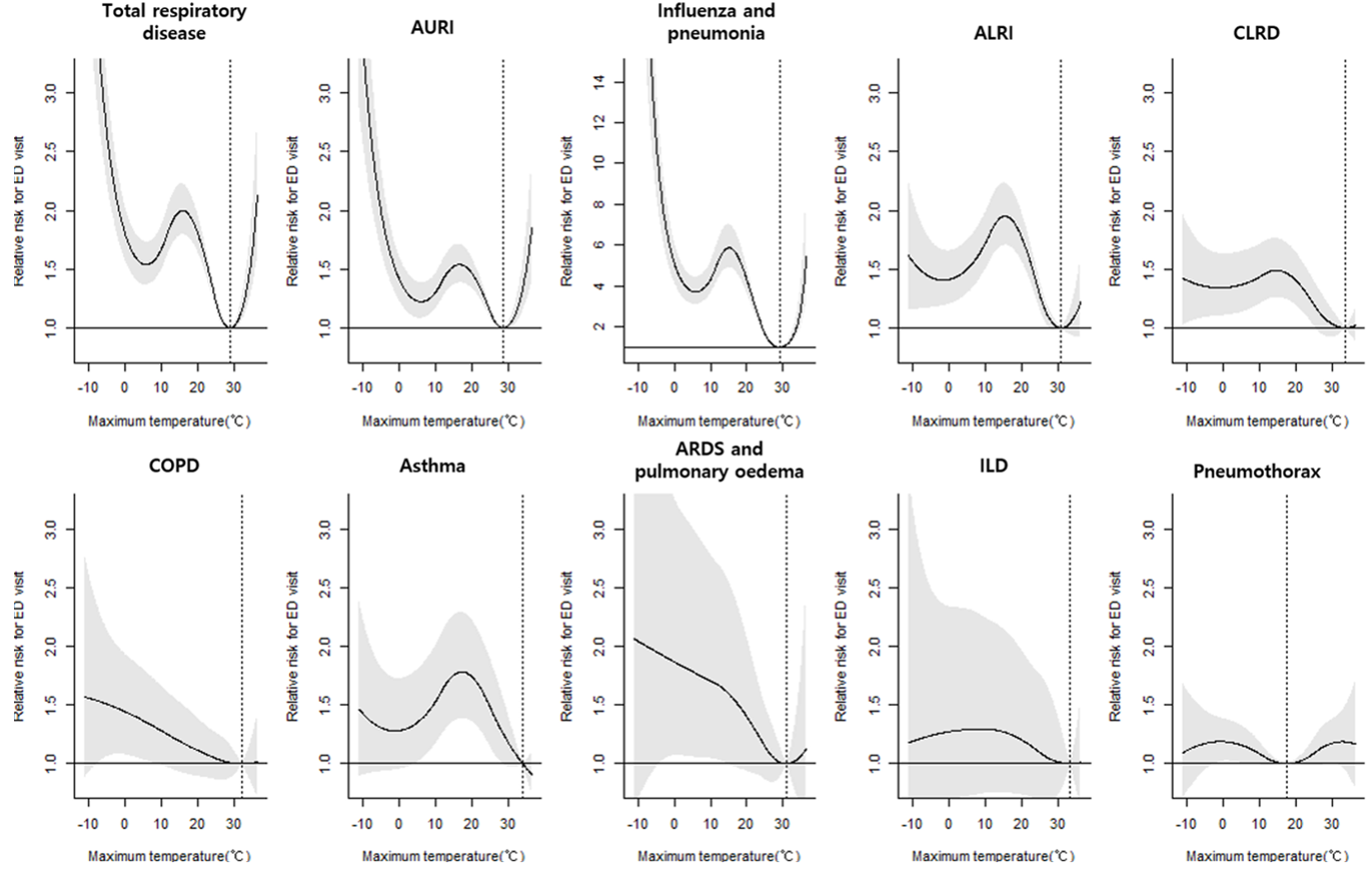


Dotted vertical lines indicate MinRT, while the solid line with shaded area represents relative risk with 95% confidence intervals.

SO_2_, sulfur dioxide; RR, relative risk; MinRT, minimum risk temperature; AURI, acute upper respiratory infection; ALRI, acute lower respiratory infection; CLRD, chronic lower respiratory disease; COPD, chronic obstructive pulmonary disease; ARDS, acute respiratory distress syndrome; ILD, interstitial lung disease

**Fig. S11.** Sensitivity analysis of the association between mean ambient temperature and emergency department visits for various subtypes of respiratory diseases, adjusted for CO concentration.


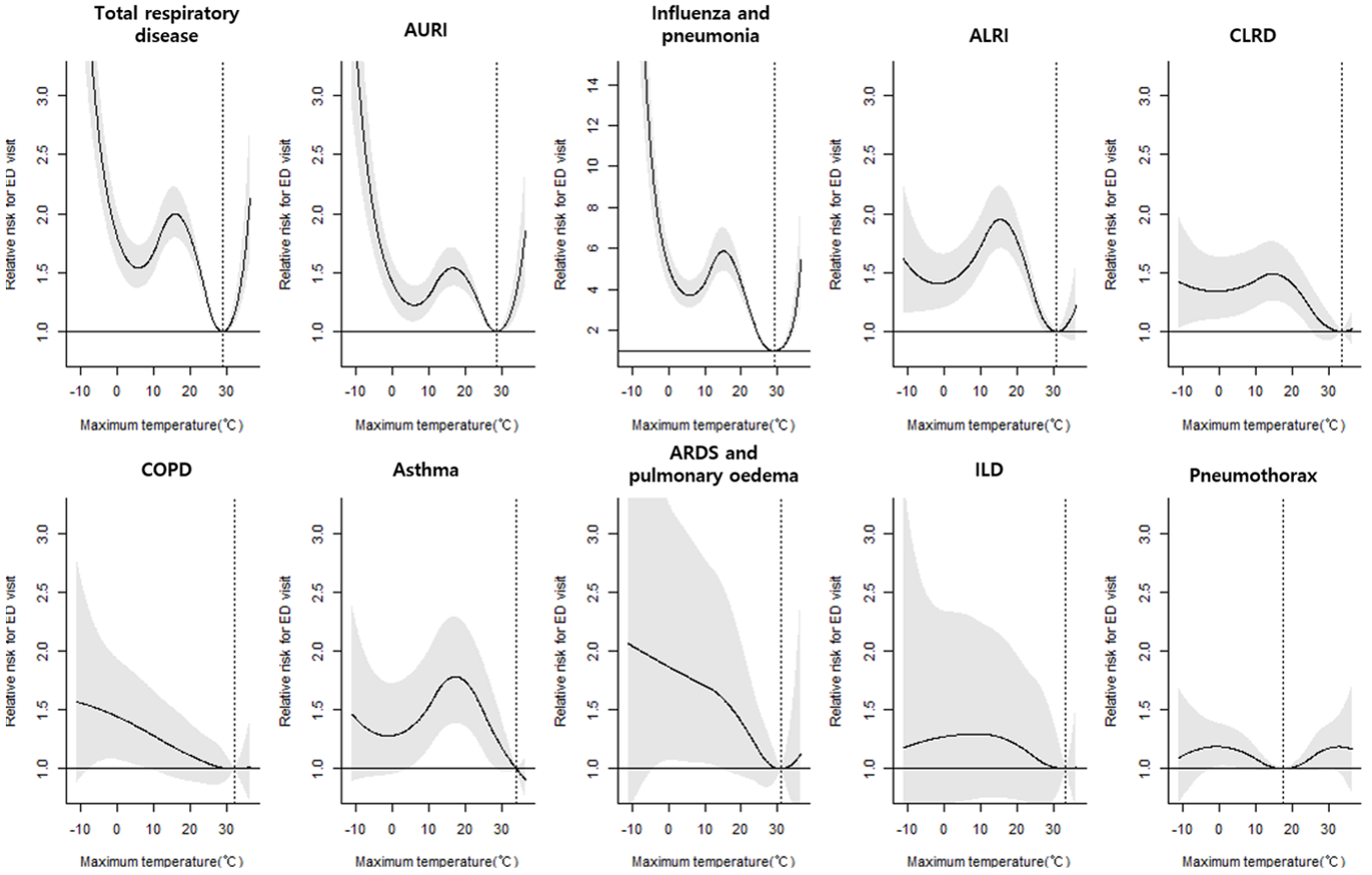


Dotted vertical lines indicate MinRT, while the solid line with shaded area represents relative risk with 95% confidence intervals.

CO, carbon monoxide; RR, relative risk; MinRT, minimum risk temperature; AURI, acute upper respiratory infection; ALRI, acute lower respiratory infection; CLRD, chronic lower respiratory disease; COPD, chronic obstructive pulmonary disease; ARDS, acute respiratory distress syndrome; ILD, interstitial lung disease
